# Real-world accuracy of SARS-CoV-2 antigen detection compared with qPCR: A cross-sectional study in Toledo - PR, Brazil

**DOI:** 10.1016/j.bjid.2025.104520

**Published:** 2025-04-17

**Authors:** Carla Adriane Royer, Regis Goulart Rosa, Maicon Falavigna, Jaqueline Carvalho de Oliveira, Toledo BNT162b2 Study Group Investigators, Mariana Motta Dias da Silva, Carolina Gracia Poitevin, Hellen Abreu, Valter Antonio Baura, Ana Claudia Bonatto, Daniela Fiori Gradia, Cristina de Oliveira Rodrigues, Rafael Messias Luiz, Ana Paula Carneiro Brandalize, Josélia Larger Manfio, Cintia Laura Pereira de Araujo, Ana Paula de Souza, Daniel Sganzerla, Caroline Cabral Robinson, Fernanda Kelly Romeiro Silva, Gabriela Almeida Kucharski, Fernando Pedrotti, Srinivas Rao Valluri, Amit Srivastava, Viviane Wal Julião, Olga Chameh Melone, Florence Lefebvre d'Hellencourt, Moe H Kyaw, Julia Spinardi, Graciela del Carmen Morales Castillo, Emanuel Maltempi de Souza

**Affiliations:** f Department of Genetics; Federal University of Parana (UFPR); Curitiba, (PR), Brazil.; g Research Unit, Inova Medical, Porto Alegre, RS, Brazil.; h Research Institute, Hospital Moinhos de Vento (HMV), Porto Alegre, RS, Brazil.; i Internal Medicine Department, HMV, Porto Alegre, RS, Brazil.; j Department of Health Research Methods, Evidence, and Impact, McMaster University, Hamilton, Ontario, Canada.; k Department of Biochemistry and Molecular Biology, UFPR, Curitiba, PR, Brazil; l Faculty of Medicine - Campus Toledo, UFPR, Brazil; m Department of Health of Toledo, Toledo, PR, Brazil; n Pfizer, Vaccines Medical and Scientific Affairs – Emerging Markets, Collegeville, PA, USA; o Orbital Therapeutics, Cambridge, MA, USA; a Department of Genetics; Federal University of Parana (UFPR); Curitiba, (PR), Brazil; b Research Unit, Inova Medical, Porto Alegre, RS, Brazil; c Research Institute, Hospital Moinhos de Vento (HMV), Porto Alegre, RS, Brazil; d Internal Medicine Department, HMV, Porto Alegre, RS, Brazil; e Department of Health Research Methods, Evidence, and Impact, McMaster University, Hamilton, Ontario, Canada

**Keywords:** COVID-19, Performance, Rapid antigen tests, Viral load

## Abstract

Rapid Antigen Tests (Ag-RDTs) for the detection of SARS-CoV-2 is an important diagnostic tool for containing virus spread. However, their accuracy ‒ the proportion of correct results (both true positives and true negatives) ‒ still needs to be proven when used in a real large-scale context. Accordingly, we provide data from a cross-sectional study conducted in Toledo - PR, Brazil, on the accuracy of rapid tests compared with qPCR within the Brazilian Unified Health System. A total of 2882 thousand individuals presenting symptoms suggestive of COVID-19 were screened. Overall, the antigen tests showed a sensitivity, specificity, and accuracy of 59 % (0.56‒0.62), 99 % (0.98‒0.99), and 82 % (0.81‒0.84) respectively. However, a significant difference was found when analysing two brand tests individually. In addition, for patients with a low quantification Cycle (Cq) < 20 (which indicates a high viral load), the agreement between test results was high (90.85 %). However, this agreement decreased significantly when the viral load decreased, dropping to 5.59 % for samples with Cq ≥ 33, which indicates a lower viral load. While rapid antigen tests are a valuable tool in combating virus spread, their accuracy can vary significantly between manufacturers and under conditions of low viral load.

SARS-CoV-2 emerged at the end of 2019 and has caused more than 775 million COVID-19 cases and over 7 million related deaths worldwide [1]. In Brazil, approximately thirty-nine million cases and more than 714,000 deaths have been registered as of January 2025 [2].

The COVID-19 pandemic has created an urgent need for rapid and accurate diagnostic tests. The two most important methods for detecting SARS-CoV-2 are Antigen-detection Rapid Diagnostic Tests (Ag-RDTs) and the gold standard of quantitative Reverse Transcription Polymerase Chain Reaction (RT-qPCR) tests [3].

Rapid antigen tests detect virus-specific proteins in respiratory specimens and offer a very short turnaround time but are generally associated with lower sensitivity. In contrast, RT-qPCR tests are more sensitive for detecting the viral genetic material but have a longer sample processing time. In addition, unlike RT-qPCR, Ag-RDTs do not require specialised infrastructure and technical expertise for point-of-care testing [4].

Although Ag-RDTs for SARS-CoV-2 have proven to be an important diagnostic tool to combat the spread of the virus, the accuracy of these tests varies greatly depending on the brand of test. In more details, accuracy value indicates the proportion of correct results, combining sensitivity (true positive rate) and specificity (true negative rate) out of the total number of tests conducted.

Recent work shows that performance values reported in real-world studies for individual SARS-CoV-2 Ag RDTs may differ from those reported in the manufacturer's instructions for use [5, 6, 7, 8, 9, 10]. Several factors, including study design and experimental bias, can influence the sensitivity estimates of Ag-RDTs; [7] therefore, monitoring their performance in practice is essential.

In this study, we present real-world data on the accuracy of two Ag-RDTs kits widely used in Brazil and compared with the gold standard RT-qPCR methods for 2882 symptomatic individuals between January 2022 and June 2022. These data were collected during a study about BNT162b2 mRNA COVID-19 against symptomatic infection following mass vaccination campaign in southern Brazil [11]. Detailed methodology and procedures were previously published [12].

In summary, our study population includes consecutive individuals aged 12-years or older who presented symptoms suggestive of COVID-19 and sought care within the public healthcare system of Toledo-PR. Two nasopharyngeal swabs were collected simultaneously. One of these swabs was analyzed using the Ag-RDTs kit, with a turnaround time of 15-minutes, while the other swab was stored in 15 mL Falcon tubes with Viral Transport Medium (VTM) at -80°C for RT-qPCR testing.

Of the 2882 Ag-RDT analyses included in the study, 2086 tests were performed with TR DPP® COVID-19 – Ag - Bio-Manguinhos (Instituto de Tecnologia em Imunobiológicos ‒ Bio-Manguinhos/Fiocruz, Rio de Janeiro, Brazil), between January and June 2022, while 796 tests were performed with the IBMP TR Covid Ag kit (Instituto de Biologia Molecular do Paraná, Curitiba, Brazil), between January and April 2022.

RNA was extracted using the Viral RNA and DNA Kit (MVXA-P096FAST, Loccus Biotecnologia, Brazil) in an automated nucleic acid extractor (Extracta 32, Loccus Biotecnologia, Brazil). Samples were then tested to confirm the presence of SARS-CoV-2 genetic material using the Centers for Disease Control and Prevention's real-time RT-PCR diagnostic protocol for 2019 novel Coronaviruses (2019-nCoV) (https://www.fda.gov/media/134922/download) on the QuantStudio™ 5 instrument (Applied Biosystems®, USA) with the GoTaq Probe 1-Step RT-qPCR system (Promega, USA).

Statistical analyses were performed using RStudio version 4.3.1. Categorical variables were presented with absolute and relative frequencies, while numerical variables were presented with mean and standard deviation. Accuracy, sensitivity, specificity, Positive Predictive Value (PPV) and Negative Predictive Value (NPV) were assessed globally and for specific categories of interest. In addition, a comparison between the categories was performed to identify any differences. The significance level was 5 %.

Using a large number of paired samples in Brazil, we evaluated the results of Ag-RDTs of two brands in comparison with RT-qPCR. The individuals tested in the community were between 12 and 87 years old, with the majority under 60 years old (92.3 %), female (57.2 %) and with up-to-date vaccination status (97.1 %). In addition, more than 88 % of people were tested between 0 and 5 days after the onset of symptoms.

Of the 2882 people included in the study, 1175 tested positive for SARS-CoV-2 by RT-qPCR (Table 1); the prevalence rate was 40.8 %. The quantification Cycle (Cq) values of the positive results ranged from 12 to 39, with most having a Cq value between 20‒25 (39.5 %), followed by ≥ 33 (27.5 %), ˂ 20 (13.0 %), 26‒28 (11.1 %) and 29‒32 (8.9 %).

**Table 1:** Comparison of Coronavirus Disease 2019 (COVID-19) rapid antigen tests (Ag-RDTs) with Reverse Transcription Polymerase Chain Reaction (RT-qPCR).

**Ag-RDTs**	**RT-qPCR**	
	**Positive**	**Negative**
General		
Positive	692 (58.9 %)	23 (1.3 %)
Negative	483 (41.1 %)	1684 (98.7 %)
IBMP TR Covid Ag kit		
Positive	389 (69.8 %)	15 (6.3 %)
Negative	168 (30.2 %)	224 (93.7 %)
TR DPP® COVID-19 ‒ Ag - Bio-Manguinhos		
Positive	303 (49.0 %)	8 (0.5 %)
Negative	315 (51.0 %)	1460 (99.5 %)

Ag-RDT, Antigen-detection Rapid Diagnostic Tests; RT-qPCR, Reverse Transcription quantitative Polymerase Chain Reaction.

The Ag-RDT showed an overall sensitivity of 59 % (0.56‒0.62). The specificity reached a value of 99 % (0.98‒0.99), and the accuracy was 82 % (0.81‒0.84). The probability of those classified as positive in the rapid test confirming their diagnosis in the RT-qPCR (positive predictive value) was 97 %, while those classified as negative confirmed the diagnosis in only 78 % of samples (negative predictive value).

The performance values of the Ag-RDTs did not differ significantly with respect to the sex, age and vaccination status of the individuals. There were no differences in the number of days after the onset of the first symptoms on which the patient was tested (Table 2).

**Table 2:** Performance of the Ag-RDTs test compared with RT-qPCR^a^.

**Variables**	**Sensitivity**	**p**	**Specificity**	**p**	**Accuracy**	**p**	**PPV**	**p**	**NPV**	**p**
Symptoms’ Days										
0–2	0.59	0.166	0.99	0.765	0.82	0.749	0.97	0.897	0.77	0.777
3–5	0.62		0.98		0.83		0.97		0.78	
6–9	0.48		0.99		0.80		0.96		0.77	
10–15	0.47		1.00		0.85		1.00		0.83	
> 15	0.62		1.00		0.88		1.00		0.85	
Vaccination status										
Vaccinated	0.59	0.384	0.99	0.989	0.83	0.144	0.97	0.987	0.78	0.070
Non-vaccinated	0.52		1.00		0.76		1.00		0.68	
Gender										
Female	0.57	0.401	0.99	0.394	0.82	0.962	0.97	0.576	0.78	0.802
Male	0.60		0.98		0.82		0.96		0.78	
Age										
< 60	0.59	0.839	0.99	0.988	0.83	0.481	0.96	0.985	0.78	0.136
≥ 60	0.60		1.00		0.81		1.00		0.73	
Test										
IBMP TR Covid Ag kit	0.70	<0.001	0.94	<0.001	0.77	<0.001	0.96	0.394	0.57	<0.001
TR DPP® COVID-19 – Ag - Bio-Manguinhos	0.49		0.99		0.85		0.97		0.82	

PPV, Positive Predictive Value; NPV, Negative Predictive Value.

a Centers for Disease Control and Prevention's real-time RT-PCR diagnostic protocol for 2019 novel coronaviruses (2019-nCoV) with the GoTaq Probe 1-Step RT-qPCR system (Promega, USA).

When comparing the SARS-CoV-2 Ag RDTs of the different manufacturers, we found significant differences in terms of sensitivity, specificity, accuracy, and negative predictive value (p-value < 0.001) (Table 2). The IBMP TR Covid Ag kit showed a sensitivity of 70 % (0.66‒0.74), while the sensitivity of the TR DPP® COVID-19 ‒ Ag - Bio-Manguinhos was 49 % (0.45‒0.53).

Viral load influenced the performance of the Ag-RDT tests. The results showed that at a high viral load, i.e., a quantification Cycle (Cq) below 20, the positivity of Ag-RDT tests matched RT-qPCR in 90.85 % of samples, but decreased with decreasing viral load, with a sensitivity of 89 % at Cq 20–25, 66 % at Cq 26–28, 34 % at Cq 29‒32 and only 5.59 % at Cq ≥ 33.

Rapid antigen tests are a valuable tool in the fight against the spread of the virus, but the accuracy of antigen tests can vary considerably, so large-scale testing of their reliability is of paramount importance for the implementation of public health strategies. Ag-RDTs have a lower sensitivity than nucleic acid amplification-based methods, which means that a lower viral load may go undetected.

The WHO recommends Ag-RDTs with a sensitivity of ≥80 % and a specificity of ≥ 97 % [4]. According to the manufacturers, the Ag-RDTs used in this study have a sensitivity of 83.3 % and a specificity of 99.7 % for the IBMP TR Covid Ag Kit and a sensitivity of 90.3 % and a specificity of 98.8 % for the TR DPP® COVID-19 – Ag - Bio-Manguinhos. However, under our real-world evidence, these values were only achieved with a high viral load, i.e., a low Cq value. The ability of the Ag-RDT to detect SARS-CoV-2 is significantly affected by viral load and is severely impaired in cases with low viral load. There is a clear inverse relationship between viral load and the concordance of positive diagnoses. Our results confirm meta-analysis and multi-center studies that have shown variations in the sensitivity of Ag-RDTs influenced by various factors such as viral load, sample quality and days after first symptoms [6–10]. Samples with a high viral load (Cq values ≤25) show a better performance of Ag-RDTs, while samples with a low viral load show a lower efficiency of these tests [6–10].

The variable sensitivity of antigen tests means that individuals who test negative may still be infected. Viral load is the most important factor in Ag-RDTs and its limitations should be considered, with viral load associated with infectivity and clearance duration [10]. However, the relationship with disease transmission is not yet clear. In the present study, the sensitivity of the Ag-RDT was found to be within the standards set by regulatory authorities for samples with high viral load. Therefore, the use of these antigen tests may be useful to isolate individuals who are more likely to transmit the infection and contribute to the management of COVID-19 spread. In uncontrolled outbreak scenarios, however, a large proportion of false-negative individuals with low viral load may contribute to the spread of the disease and may justify a different approach to Burden of Disease management.

## Conflicts of interest

The authors declare no conflicts of interest.
